# Reduced complex I activity in the retinal pigment epithelium, but not in rod photoreceptors, affects light signaling without impacting cell survival

**DOI:** 10.1016/j.jbc.2025.110586

**Published:** 2025-08-12

**Authors:** Alexander M. Warwick, Lin Yu, Mikael Klingeborn, Amanda M. Travis, Vipul M. Parmar, Howard M. Bomze, Goldis Malek, Sidney M. Gospe III

**Affiliations:** 1 Department of Ophthalmology, Duke University School of Medicine, Durham, North Carolina, USA; 2 Department of Pharmaceutical Sciences, University of Colorado Anschutz Medical Campus, Aurora, Colorado, USA; 3 Department of Pathology, Duke University School of Medicine, Durham, North Carolina, USA

**Keywords:** photoreceptor, retina, retinal pigment epithelium, electrophysiology, complex I, mitochondrial disease

## Abstract

Mutations in the mitochondrial respiratory complex I accessory subunit NADH:ubiquinone oxidoreductase subunit S4 (*ndufs4)* can cause the mitochondrial disease Leigh syndrome, which may be associated with vision loss. We previously demonstrated that mice with global deletion of *ndufs4* exhibited impaired *in vivo* photoreceptor light responses prior to the early death of the mice around postnatal day 50. However, *ex vivo* electrophysiology recordings performed on retinas from *ndufs4*^*−/−*^ mice were normal, suggesting that the *in vivo* phenotype may reflect altered homeostasis of the extracellular environment of photoreceptors rather than their intrinsic metabolic dysfunction. To test this hypothesis, we have generated mouse strains with cell-specific deletions of *ndufs4* from rod photoreceptors and from the retinal pigment epithelium (RPE), a key supporting cell to photoreceptors. We now demonstrate that despite efficient depletion of NDUFS4 protein and consequent reduction of complex I activity in rods, scotopic electroretinography (ERG) responses are essentially normal and rod survival is not impacted by rod-specific *ndufs4* deletion. Interestingly, while RPE-specific deletion of *ndufs4* depletes NDUFS4 protein and reduces complex I activity in RPE, a ∼15% reduction in ERG amplitudes is observed, much less than the 50% reduction previously reported in global *ndufs4*^*−/−*^ mice. This suggests that a more complex metabolic relationship exists between photoreceptors, RPE, and other cells of the retina to establish the homeostatic physiological conditions necessary for normal light signaling.

Mitochondria house the enzymatic machinery for oxidative phosphorylation (OXPHOS), the chief means of ATP production for most eukaryotic cells. The retina, one of the most metabolically active tissues of the body, is highly sensitive to mitochondrial dysfunction (1). For instance, mitochondrial pathology has been implicated in diseases of the outer retina and retinal pigment epithelium (RPE), such as pigmentary retinopathy and age-related macular degeneration (2, 3, 4). However, despite experiencing great bioenergetic demands under both darkness and illumination, retinal photoreceptors are unusually dependent on aerobic glycolysis rather than OXPHOS for ATP generation, a phenomenon known as the Warburg effect (5, 6). Accordingly, other cell types—particularly the RPE—are thought to be the primary drivers of outer retinal diseases related to mitochondrial dysfunction (7, 8).

The RPE is a cellular monolayer that separates the neural retina from the choroidal circulation and forms part of the blood–retina barrier. It is responsible for delivery of metabolic substrates from the circulation to the photoreceptors, most notably glucose. The facilitative glucose transporter GLUT1 exists on both the basolateral and apical membranes of the RPE (9), providing a direct route for delivery of glucose to the subretinal space, where it is taken up by photoreceptors *via* GLUT1 on the plasma membrane of photoreceptor inner segments (10). It has been proposed by Hurley et al that photoreceptors and the RPE exist in a symbiotic “metabolic ecosystem” in which photoreceptors release metabolic intermediates such as lactate and succinate to serve as substrates for oxidative metabolism by the RPE; in turn, the RPE spares glucose derived from the choroidal circulation, allowing it to be delivered to photoreceptors for use in glycolysis (11, 12). It follows from this model that decreased OXPHOS capacity could make the RPE more reliant on glycolysis for its own energy needs, thus starving photoreceptors of energy substrate. Indeed, impaired glucose delivery from the RPE to photoreceptors has been suggested as a key contributor to vision loss in retinitis pigmentosa and age-related macular degeneration (3, 13).

Our previous analysis of mice with severe mitochondrial complex I impairment provided evidence potentially supporting this concept. We characterized abnormal retinal light signaling in mice lacking NADH:ubiquinone oxidoreductase subunit S4 (NDUFS4), a key accessory subunit of complex I (14). KO of *ndufs4* has been reported to result in severe instability of complex I, reducing its enzymatic activity by >50% in the retina and other tissues (15, 16, 17). We observed abnormal rod and cone light responses in electroretinography (ERG) recordings performed on live *ndufs4*^*−/−*^ mice, with a- and b-wave amplitudes decreased by approximately 50% under both scotopic and photopic conditions. However, within the short lifespan of these mutant mice (∼50 days), we found no evidence of photoreceptor degeneration or of any developmental or morphological anomalies. Strikingly, *ex vivo* ERG recordings conducted on isolated *ndufs4*^*−/−*^ retinas revealed normal light responses, suggesting that the impaired light responses in live mice were not due to irreversible photoreceptor dysfunction, but instead likely originated from abnormal outer retinal homeostasis that may be overcome by bathing retinas in nutrient-rich culture media.

Here, we describe a series of experiments designed to determine the relative importance of OXPHOS within the photoreceptors and RPE in supporting normal outer retinal signaling. Rod-specific complex I deficiency produced a negligible *in vivo* signaling phenotype, supporting our hypothesis that intact OXPHOS is dispensable for normal light responses by photoreceptors. Conversely, RPE-specific complex I dysfunction produced a significant, albeit smaller-than-expected, decrease in ERG amplitudes, demonstrating that the RPE plays at least a partial role in maintaining the metabolic environment necessary for optimal photoreceptor light signaling. The more pronounced signaling abnormalities previously observed in mice with global complex I deficiency are likely a reflection of additional cell types contributing to outer retinal metabolic homeostasis.

## Results

### NDUFS4 is efficiently depleted from the rods of iCre75;ndufs4^loxP/loxP^ mice and results in reduced retinal complex I activity

**Figure 1:**
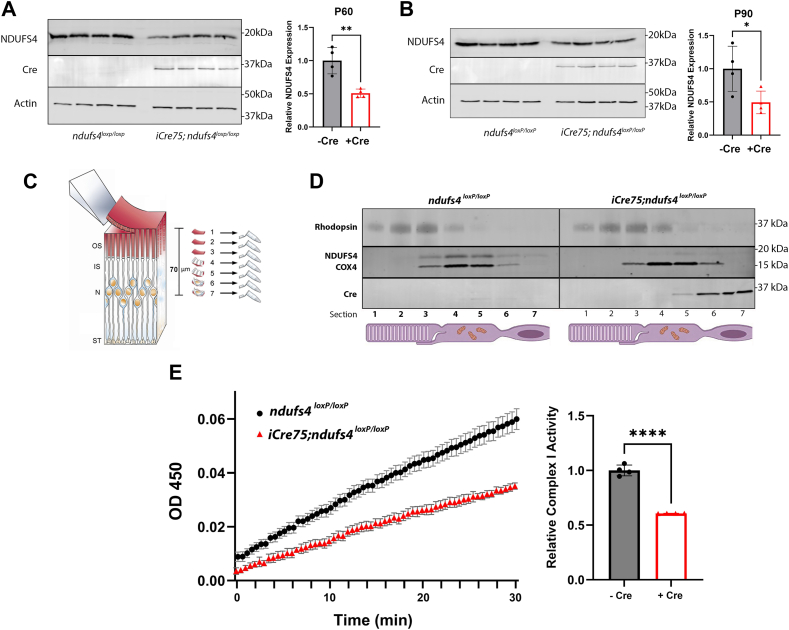
**NDUFS4 protein is efficiently depleted from rod photoreceptors of *iCre75*;*ndufs4*^*loxP/loxP*^ mice**. *A* and *B*, western blot analyses of lysates from whole retinas of four *iCre75*;*ndufs4*^*loxP/loxP*^ mice and four *ndufs4*^*loxP/loxP*^ littermate control mice at P60 (*A*) and P90 (*B*). The intensity of each NDUFS4 protein band was normalized to that of each corresponding actin loading control band, and the ratio of NDUFS4 abundance in *iCre75*;*ndufs4*^*loxP/loxP*^ retinas to that of control retinas is shown in the graphs to the *right*. Data depicted as mean ± standard deviation, with biological replicates shown as individual data points; ∗, *p* ≤ 0.05; ∗∗, *p* ≤ 0.01 by two-tailed *t* test. *C*, schematic illustration of serial tangential sectioning of a retinal flat mount. Ten micrometer sections were sequentially obtained, with photoreceptor outer segment (OS) material enriched in sections 1 to 3, inner segment (IS) material in sections 4 to 5, and the nuclear (N) material in sections 6 to 7. Image modified with permission from (41). *D*, western blot analysis of serial sections obtained from the outer retinas of a *ndufs4*^*loxP/loxP*^ mouse (*left*) and an *iCre75*;*ndufs4*^*loxP/loxP*^ mouse (*right*). The bands for NDUFS4 and another mitochondrial protein, COX4, are shown in the *middle row*. *E*, colorimetric assay of respiratory complex I activity in whole retinal lysates from four *iCre75*;*ndufs4*^*loxP/loxP*^ mice and four *ndufs4*^*loxP/loxP*^ littermates. The complex I activity of *iCre75*;*ndufs4*^*loxP/loxP*^ retinas relative to *ndufs4*^*loxP/loxP*^ control retinas is shown in the graph to the *right*. Data depicted as mean ± standard deviation, with biological replicates shown as individual data points; ∗∗∗∗, *p* ≤ 0.0001 by two-tailed *t* test. NDUFS4, NADH:ubiquinone oxidoreductase subunit S4; COX4, Cytochrome c Oxidase Subunit 4.

We previously observed no evidence of rod photoreceptor degeneration or morphological abnormalities in *ndufs4*^*−/−*^ mice despite a marked reduction of *in vivo* ERG amplitudes, including the scotopic a-wave that arises exclusively from rods (18). However, because the global impairment of complex I activity in *ndufs4*^*−/−*^ mice results in rapid lethality beginning at P50, a slower developing degenerative phenotype in rods could not be ruled out. Therefore, to investigate the importance of intrinsic complex I function to rod function and survival, we created a model of rod-specific deletion of *ndufs4* by crossing a mouse line carrying *floxed* alleles of *ndufs4* (*ndufs4*^*loxP/loxP*^) (15) with the *iCre75* mouse strain which expresses rhodopsin-driven Cre recombinase in the vast majority of rods and in no other retinal neurons (19). Because rods comprise ∼80% of all retinal cells (20), loss of NDUFS4 protein from virtually all rods was expected to be detectable on western blot analysis of whole retinal lysates. We observed that at P60, retinas from *iCre75*;*ndufs4*^*loxP/loxP*^ mice exhibited a reduction of NDUFS4 protein content to 51% that of *ndufs4*^*loxP/loxP*^ littermates lacking Cre recombinase [95% confidence interval: (0.41, 0.61)] (Fig. 1*A*). Cre recombinase expression in *iCre75* mice begins around P7 (19), and the lifetime of NDUFS4 in rods has not been reported. Therefore, it was unclear to what extent rod-derived NDUFS4 protein had been depleted by P60. Upon repeating the experiment at the later time point of P90, we observed a similar reduction of NDUFS4 protein from whole retina to 49% [95% confidence interval: (0.22, 0.76)], indicating that maximal depletion had likely already occurred at the P60 time point. (Fig. 1*B*).

Although the effectiveness of the *iCre75* strain in achieving Cre-mediated recombination ubiquitously in rods is very well established, it was important to confirm that the ∼50% of NDUFS4 content remaining was coming from the non-rod cellular population of *iCre75*;*ndufs4*^*loxP/loxP*^ retinas. Accordingly, we obtained serial 10-μm-thick tangential sections through flat-mounted P90 retinas to profile the expression of NDUFS4 throughout the outermost retinal layers, which are comprised almost entirely by photoreceptors (Fig. 1, *C* and *D*). The inner segment layer of the retina (represented predominantly in sections 4 and 5) is rich in mitochondria, as indicated by abundant expression of NDUFS4 and the complex IV subunit COX4 in the control *ndufs4*^*loxP/loxP*^ retina (Fig. 1*D*). In contrast, the adjacent outer segment layer (marked by rhodopsin, primarily in sections 1-3) and outer nuclear layer (starting in sections 6 and 7) are known to be devoid of mitochondria. Whereas COX4 protein remained in normal abundance in the inner segments of *iCre75*;*ndufs4*^*loxP/loxP*^ photoreceptors, there was a near-complete loss of NDUFS4 protein in these same sections (Fig. 1*D*). This suggests that virtually 100% of NDUFS4 protein is depleted from rods, with the minor fraction of remaining NDUFS4 signal in these sections likely coming from cone photoreceptors and the apical region of Müller glia, neither of which express Cre recombinase in this model. Notably, as a nuclear-targeted protein (21), Cre recombinase effectively marks the outer nuclear layer in *iCre75*;*ndufs4*^*loxP/loxP*^ retinal sections (Fig. 1*D*).

Because loss of NDUFS4 in the global *ndufs4* KO mouse has been reported to reduce complex I activity by >50% in the retina (17), we expected that the ∼50% reduction of NDUFS4 protein content from the retinas of *iCre75*;*ndufs4*^*loxP/loxP*^ mice would have a detectable effect on complex I activity from whole retinal lysates. Indeed, we observed that the complex I activity of retinas from P90 *iCre75*;*ndufs4*^*loxP/loxP*^ mice was reduced to 61% [95% confidence interval: (0.605, 0.609)] compared to *ndufs4*^*loxP/loxP*^ littermates with NDUFS4 intact in rods (Fig. 1*E*).

### Loss of NDUFS4 has minimal impact on rod light signaling and does not affect survival

**Figure 2:**
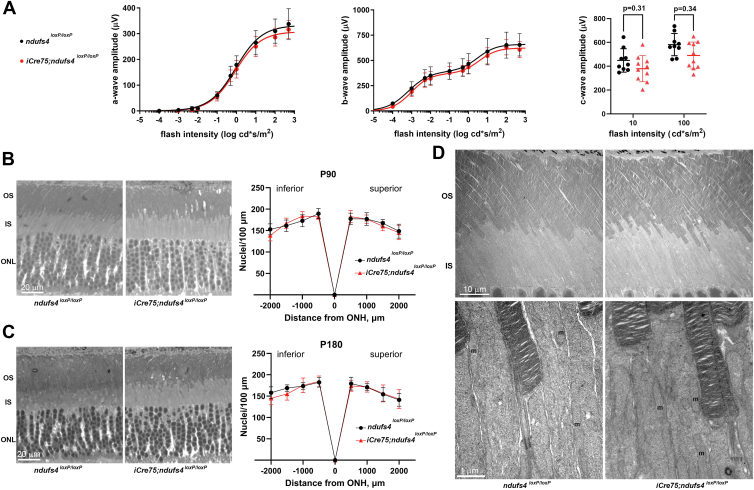
**Reduced complex I activity in rod photoreceptors does not induce meaningful changes in light responses or lead to rod degeneration**. *A*, ERG responses of dark-adapted P90 *iCre75*;*ndufs4*^*loxP/loxP*^ mice and *ndufs4*^*loxP/loxP*^ littermate controls (n = 10 eyes of five mice per genotype) are plotted as a function of flash intensity and fit using a single or double hyperbolic function. The *left panel* shows scotopic a-wave amplitudes and the *middlepanel* shows scotopic b-wave amplitudes. The *right panel* depicts the c-wave amplitudes recorded for each group at stimulus intensities of 10 and 100 cd∗s/m^2^, with biological replicates shown as individual data points. All data are presented as mean ± standard deviation. For both a- and b-wave amplitudes, statistical comparison between each group by two-tailed *t* test failed to demonstrate differences achieving a significance level of *p* < 0.05 for any flash intensity tested. *B* and *C*, representative cross sections of a *ndufs4*^*loxP/loxP*^ control retina (*left*) and an *iCre75*;*ndufs4*^*loxP/loxP*^ retina (*right*) at P90 (*B*) and P180 (*C*). The number of photoreceptor nuclei within 100 μm segments of the outer nuclear layer were quantified at 500 μm intervals inferiorly and superiorly from the optic nerve head (ONH) and plotted in spider diagrams to the *right* at each time point. Graphs depict mean ± standard deviation. No statistical difference in the nuclear counts was observed between the two genotypes at any of the locations or at either time point by two-tailed *t* test. n = 5 retinas per genotype at P90 and 6 retinas per genotype at P180. OS, outer segment; IS, inner segment; ONL, outer nuclear layer. *D*, representative electron micrographs of photoreceptors from a control *ndufs4*^*loxP/loxP*^ mouse and a littermate *iCre75*;*ndufs4*^*loxP/loxP*^ mouse at P180. The *top panels* depict well-ordered arrays of outer segments in both genotypes, while the bottom higher-magnification images show the apical region of photoreceptor inner segments, with several examples of mitochondria labeled (m). ERG, electroretinography.

Having confirmed effective depletion of NDUFS4 protein from the rods of *iCre75*;*ndufs4*^*loxP/loxP*^ mice, we proceeded to determine whether the rod-specific deletion of *ndufs4* would impair scotopic ERG responses in these mice. Dark-adapted P90 *iCre75*;*ndufs4*^*loxP/loxP*^ mice and *ndufs4*^*loxP/loxP*^ littermate controls (n = 5 for each genotype) underwent ERG recordings using 10 separate stimuli of progressively higher intensity (Fig. 2*A*). Unlike the global *ndufs4* KO mouse, in which we had previously observed ∼50% amplitude reductions of both the a-wave (derived from photoreceptors) and b-wave (derived from inner retinal neurons in response to photoreceptor signaling) (14), the *iCre75*;*ndufs4*^*loxP/loxP*^ mice showed no meaningful change in their *in vivo* ERG responses compared to *ndufs4*^*loxP/loxP*^ controls. Although the fitted curves for the a-waves of each genotype were statistically different (*p* = 0.033; F-test), there was no significant difference between the response amplitudes at any of the 10 stimulus intensities tested (Fig. 2*A*). Furthermore, there was no statistical difference in the fitted curves for the b-wave amplitudes (*p* = 0.268; F-test). The failure of rod-specific *ndufs4* deletion to phenocopy the electrophysiological abnormalities observed in the global *ndufs4*^*−/−*^ mice was consistent with our hypothesis that impaired photoreceptor signaling in *ndufs4*^*−/−*^ mice was a consequence of changes to the local microenvironment rather than to the intrinsic complex I dysfunction of the photoreceptors. We also recorded c-waves at two stimulus intensities (10 and 100 cd‧s/m^2^) as a measure of electrical activity arising primarily from the RPE (22, 23, 24); as expected, rod-specific deletion of *ndufs4* did not significantly affect c-wave amplitudes (Fig. 2*A*).

To assess for any histological phenotype arising from rod-specific loss of NDUFS4, plastic cross-sections were prepared from posterior eyecups obtained from the P90 *iCre75*;*ndufs4*^*loxP/loxP*^ mice and *ndufs4*^*loxP/loxP*^ littermate controls. Nuclei were counted in the outer nuclear layer at multiple distances from the optic nerve head to quantify the number of surviving photoreceptors. Despite complete loss of NDUFS4 protein and the consequent reduction of complex I activity, photoreceptor abundance was unaffected in mice with rod-specific *ndufs4* deletion (Fig. 2*B*). To investigate the possibility of a slower-developing degeneration of rods, we also quantified outer nuclear layer nuclei at P180 and similarly found no significant reduction of photoreceptor nuclei in the *iCre75*;*ndufs4*^*loxP/loxP*^ mice at this later age (Fig. 2*C*). The P180 retinas were also evaluated for ultrastructural abnormalities by electron microscopy (Fig. 2*D*), and the *ndufs4*-deficient rods demonstrated normal outer segments with well-ordered flattened discs, indistinguishable from those of control littermates. The mitochondria of the inner segments also had normal morphology with no evidence of swelling or loss of cristae.

### Induction of RPE-specific Cre recombinase activity depletes NDUFS4 protein and reduces complex I activity in RPE65-CreER^T2^ndufs4^loxP/loxP^ mice

Having demonstrated that an intrinsic oxidative phosphorylation deficiency of rods does not affect light signaling, we next turned to the RPE. In light of the key roles attributed to the RPE in providing metabolic support to photoreceptors, we hypothesized that RPE-specific loss of NDUFS4 would phenocopy the electrophysiological abnormalities of global *ndufs4*^*−/−*^ mice. Although a number of mouse strains exist that express Cre recombinase specifically in RPE, most of them exhibit substantial mosaicism (25, 26, 27, 28). The presence of patches of RPE with normal metabolism would be likely to mask any secondary effect of RPE-specific complex I dysfunction on photoreceptor physiology mediated by alterations of the extracellular environment. Therefore, we elected to use the recently developed *RPE65-CreER*^*T2*^ mouse line in which RPE-specific Cre activity is achieved in >99% of RPE cells after induction by five consecutive days of intraperitoneal injections of tamoxifen (29).

**Figure 3:**
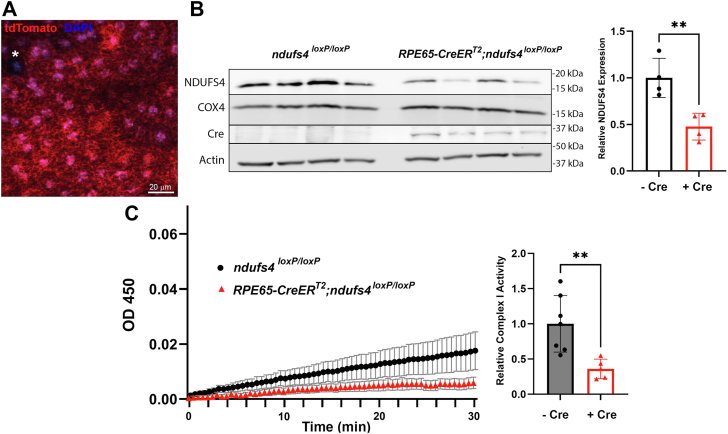
**RPE-specific depletion of NDUFS4 protein leads to reduced complex I activity**. *A*, representative RPE flat mount of a *RPE65-CreER*^*T2*^;*Ai9* mouse demonstrating expression of the tdTomato Cre reporter (*red*) in nearly all RPE cells following induction of Cre activity with tamoxifen. RPE nuclei are labeled with DAPI (*blue*). A single RPE cell lacking tdTomato expression is marked with an *asterisk* (∗). *B*, western blot analysis of enriched RPE preparations from the posterior eyecups of four *RPE65-CreER*^*T2*^;*ndufs4*^*loxP/loxP*^ mice and four *ndufs4*^*loxP/loxP*^ littermate control mice at P150 following intraperitoneal injection of tamoxifen for 5 days starting at P22. The intensity of each NDUFS4 protein band was normalized to that of each corresponding actin loading control band, and the ratio of NDUFS4 abundance in *RPE65-CreER*^*T2*^;*ndufs4*^*loxP/loxP*^ samples to that of control samples is depicted in the graph to the *right*. ∗∗, *p* ≤ 0.01 by two-tailed *t* test. *C*, colorimetric assay of respiratory complex I activity in RPE/choroid lysates from tamoxifen-induced *RPE65-CreER*^*T2*^;*ndufs4*^*loxP/loxP*^ mice (n = 5) and *ndufs4*^*loxP/loxP*^ littermates (n = 7) at P150. The complex I activity of *RPE65-CreER*^*T2*^;*ndufs4*^*loxP/loxP*^ samples relative to control is shown in the graph to the *right*. Biological replicates are shown as individual data points, and the bar graph depicts mean ± standard deviation; ∗∗, *p* ≤ 0.01 by two-tailed *t* test. RPE, retinal pigment epithelium; DAPI, 4′,6-diamidino-2-phenylindole; NDUFS4, NADH:ubiquinone oxidoreductase subunit S4.

To confirm sufficient efficiency of Cre-mediated recombination in the RPE, we crossed the *RPE65-CreER*^*T2*^ mice with the *Ai9* reporter line in which the fluorescent protein tdTomato is expressed in a Cre-dependent manner (30). By assessing tdTomato expression in RPE flat mounts (Fig. 3A), we found that nearly all RPE of *RPE65-CreER*^*T2*^;*Ai9* mice administered tamoxifen 75 mg/kg from P22 to 26 exhibited Cre-mediated recombination at P120 (99.9%; n = 3397 RPE cells counted in four retinas). We then proceeded to generate *RPE65-CreER*^*T2*^;*ndufs4*^*loxP/loxP*^ mice and control *ndufs4*^*loxP/loxP*^ littermates lacking Cre. All mice were treated with tamoxifen from P22 to P26, and posterior eyecup tissue enriched for RPE was harvested at P150 for quantification of NDUFS4 protein content. After performing western blot analysis of lysates from this tissue and normalizing NDUFS4 band intensities to that of actin we observed a reduction of NDUFS4 protein content in *RPE65-CreER*^*T2*^;*ndufs4*^*loxP/loxP*^ mice to 48% [95% confidence interval: (0.25, 0.70)] compared to controls (Fig. 3*B*). The effects of RPE-specific *ndufs4* deletion on the complex I activity of RPE/choroid lysates was quite pronounced, with a reduction to 36% [95% confidence interval: (0.24, 0.48)] in the *RPE65-CreER*^*T2*^;*ndufs4*^*loxP/loxP*^ mice compared to *ndufs4*^*loxP/loxP*^ controls (*p* ≤ 0.01; Fig. 3*C*).

### RPE-specific deletion of ndufs4 produces a mild impairment of retinal light responses and does not lead to RPE or photoreceptor degeneration

**Figure 4:**
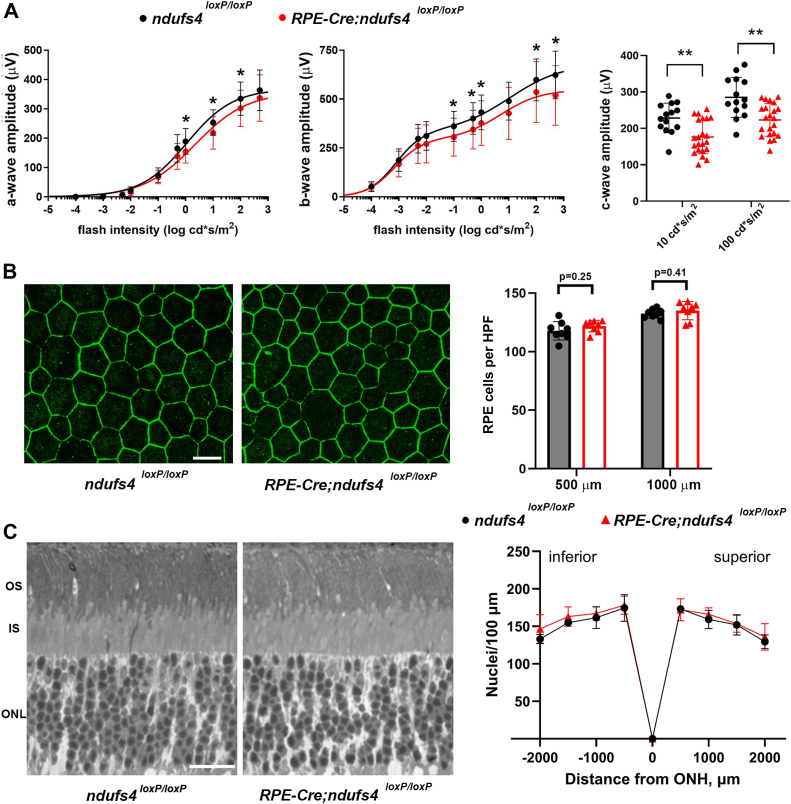
**Reduced complex I activity in RPE leads to a mild decrease in light signaling in the absence of degeneration of RPE or photoreceptors**. *A*, ERG responses of tamoxifen-induced, dark-adapted P150 *RPE65-CreER*^*T2*^;*ndufs4*^*loxP/loxP*^ mice (n = 14 eyes of seven mice) and *ndufs4*^*loxP/loxP*^ littermate controls (n = 10 eyes of five mice). The *left panel* and *middle panel* depict the amplitudes of the scotopic a-waves and b-waves, respectively, plotted as a function of flash intensity and fit using a single or double hyperbolic function. The *right panel* depicts the c-wave amplitudes recorded at stimulus intensities of 10 and 100 cd∗s/m^2^, with biological replicates depicted as individual data points. Data for each flash intensity are presented as mean ± standard deviation. ∗, *p* ≤ 0.05; ∗∗, *p* ≤ 0.01 by two-tailed *t* test. *B*, representative RPE flat mounts of tamoxifen-induced *ndufs4*^*loxP/loxP*^ and *RPE65-CreER*^*T2*^;*ndufs4*^*loxP/loxP*^ mice at P150, immunolabeled for ZO-1 (*green*) to delineate cell-cell junctions. The scale bar represents 20 μm. The graph to the *right* depicts mean RPE cell counts from *ndufs4*^*loxP/loxP*^ mice (*gray*) and *RPE65-CreER*^*T2*^;*ndufs4*^*loxP/loxP*^ mice (*red*) within 45,000 μm^2^ high power fields, obtained at locations 500 μm and 1000 μm from the optic nerve head. Error bars depict standard deviation, n = 8 flat mounts per genotype, with each biological replicate depicted as individual data points. *C*, representative cross sections of retinas from a tamoxifen-induced *ndufs4*^*loxP/loxP*^ control mouse (*left*) and *RPE65-CreER*^*T2*^;*ndufs4*^*loxP/loxP*^ mouse (*right*) at P150. OS, outer segment; IS, inner segment; ONL, outer nuclear layer. The scale bar represents 10 μm. The number of photoreceptor nuclei within 100 μm segments of the outer nuclear layer were quantified at 500 μm intervals inferiorly and superiorly from the optic nerve head (ONH) and plotted in the spider diagram to the *right*. Data are depicted as mean ± standard deviation. No significant difference in the nuclear counts was observed between the two genotypes at any of the locations by two-tailed *t* test. n = 8 retinas per genotype. ERG, electroretinography; RPE, retinal pigment epithelium.

We next characterized the impact of RPE-specific complex I dysfunction on retinal light signaling. ERGs were performed on dark-adapted *RPE65-CreER*^*T2*^;*ndufs4*^*loxP/loxP*^ mice and control *ndufs4*^*loxP/loxP*^ littermates at P150, following tamoxifen induction from P22 to 26 (Fig. 4*A*). We observed a 20 to 25% reduction of the c-wave amplitudes recorded at the 10 and 100 cd‧s/m^2^ stimulus intensities (*p* < 0.01 for both), consistent with abnormal electrical activity of RPE with impaired oxidative metabolism. Turning to retinal neuron function, in contrast to the *iCre75*;*ndufs4*^*loxP/loxP*^ mice with rod-specific *ndufs4* deletion, the *RPE65-CreER*^*T2*^;*ndufs4*^*loxP/loxP*^ mice demonstrated a clear reduction of both a-wave and b-wave amplitudes (*p* < 0.0001 for both by F-test; Fig. 4*A*). However, the observed amplitude reductions of approximately 15% for both the a- and b-waves were much more modest than the ∼50% impairment we previously observed in global *ndufs4*^*−/−*^ mice (14).

It was possible that the relatively mild ERG abnormalities observed with RPE-specific NDUFS4 depletion could be a reflection of the onset of a degenerative process, so we performed a histological characterization of the RPE and photoreceptors from the *RPE65-CreER*^*T2*^;*ndufs4*^*loxP/loxP*^ mice. However, the density and morphology of the RPE at P150 did not differ from control littermates (Fig. 4*B*), and photoreceptor nuclei counts in the outer nuclear layer were also unaffected, indicating that no photoreceptor degeneration had occurred (Fig. 4*C*). Therefore, while milder than the phenotype of global *ndufs4*^*−/−*^ mice, reduced complex I activity in the RPE appears to impact the electrophysiology of photoreceptors without producing overt degeneration.

**Figure S1:**
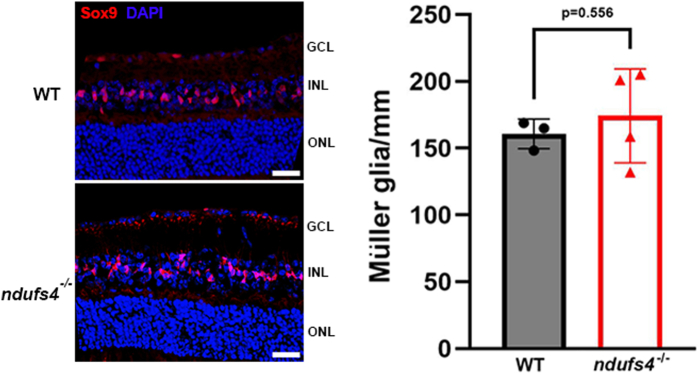
**Figure S1. Quantification of Müller glia abundance in *ndufs4* global knockout mouse retinas.** Left, representative images of cross sections of wild type (WT) and *ndufs4^-/-^* mouse retinas, with the cell bodies of Müller glia labeled with Sox9 (red) and the outer nuclear layer (ONL), inner nuclear layer (INL), and ganglion cell layer (GCL) labeled with DAPI (blue). The scale bars indicate 25 μm. Right, comparison of the abundance of Müller glia cell bodies per micron length of INL in WT and *ndufs4^-/-^* retinas. N=3 WT mice and 4 *ndufs4^-/-^* mice. Biological replicates are shown as individual data points. Error bars depict standard deviation.

The relatively mild reduction of ERG a- and b-wave amplitudes in *RPE65-CreER*^*T2*^;*ndufs4*^*loxP/loxP*^ mice compared to the *ndufs4*^*−/−*^ strain with global impairment of OXPHOS suggests that dysfunction of other retinal cell types may also contribute to the outer retinal signaling deficiency of *ndufs4*^*−/−*^ mice. The most logical candidate is Müller glia, which play key supporting roles for retinal neurons *via* extracellular electrolyte buffering, neurotransmitter recycling, and exchange of metabolic intermediates (31). To determine if degeneration of Müller glia might account for the previously observed reduction of ERG amplitudes in *ndufs4*^*−/−*^ mice, we quantified the cell bodies of Müller glia in the inner nuclear layer of P40 *ndufs4*^*−/−*^ retinas by immunolabeling for Sox9 (Fig. S1). However, no difference in the abundance of Müller glia was noted compared to WT controls.

## Discussion

Our electrophysiological evaluation of mice with cell-specific loss of the complex I accessory subunit NDUFS4 revealed that intrinsic complex I deficiency did not meaningfully impact rod light signaling. However, decreased complex I activity in the RPE partially impaired retinal signaling. Similar to our prior findings in an analysis of *ndufs4*^*−/−*^ mice in which global complex I dysfunction did not induce any early photoreceptor degeneration by P47 (14), we did not detect any loss of photoreceptors or morphological abnormalities after several additional months of rod-specific NDUFS4 depletion (up to P180). This also held true in the case of RPE-specific loss of NDUFS4, with neither photoreceptors nor RPE exhibiting signs of degeneration 4 months after Cre-mediated deletion of the gene. The lack of cellular degeneration is in stark contrast to our observation of a severe progressive degeneration of retinal ganglion cells deficient in NDUFS4 (18), indicating substantial variability of the sensitivity of different retinal cell populations to mitochondrial impairment.

In the case of rod-specific complex I dysfunction, the absence of an electrophysiological phenotype was not particularly surprising. Our interpretation of our previous observations of decreased *in vivo*, but not *ex vivo*, retinal light responses in the setting of global *ndufs4* deletion was that decreased OXPHOS within photoreceptors does not impact their ability to signal as long as their extracellular environment provides the necessary metabolic support. The apparent dispensability of oxidative metabolism to normal photoreceptor physiology is in keeping with reports that the partial pressure of O_2_ is naturally very low in the outer retina (5- to 10-fold lower than in the RPE or inner retina (32, 33)). Rather, it seems that other metabolic pathways such as glycolysis are sufficient to power phototransduction (as indicated by normal a-wave amplitudes) and synaptic communication with second order retinal neurons (demonstrated by normal b-wave amplitudes).

The aphenotypic nature of the rod-specific *ndufs4* deletion may have some implications for the metabolic ecosystem model proposed by Hurley et al (11, 12), in that it may give clues to the relative importance of particular metabolic substrates provided by photoreceptors to the RPE in exchange for glucose. With rod-specific *ndufs4* deletion, highly active photoreceptor glycolysis could continue to generate ample lactate to be passed to the RPE, converted back to pyruvate and used as a substrate for OXPHOS. However, impairment of photoreceptor complex I would be predicted to be detrimental to the provision of succinate by photoreceptors to the RPE. In Hurley’s model, the passage of electrons from complex I to coenzyme Q_10_ is needed so that complex II (succinate dehydrogenase) may run in reverse to generate succinate from fumarate. Indeed, it was demonstrated that retinas from global *ndufs4*^*−/−*^ mice had a reduced succinate pool due to increased forward and reduced reverse complex II activity (12). However, if a large flux of succinate from photoreceptors to the RPE is required in order for the RPE to spare choroid-derived glucose for use by photoreceptors, then one might have expected our model of complex I-deficient rods (which represent >97% of photoreceptors in mice (20)) to exhibit a pronounced ERG phenotype due to glucose starvation. A future analysis of the overall content and rate of release of lactate, succinate, and other metabolites from the retinas of *iCre75*;*ndufs4*^*loxP/loxP*^ mice might help to elucidate the relative contributions and potential interchangeability of these compounds in the metabolic relationship between photoreceptors and the RPE.

The significant but smaller-than-expected impact of RPE-specific loss of NDUFS4 on retinal signaling is interesting. Without causing overt photoreceptor degeneration, the decrease in RPE complex I activity consistently resulted in a ∼15% reduction of both a- and b-wave amplitudes, indicative of an indirect impact on the efficiency of phototransduction by photoreceptors. We had hypothesized that the induction of RPE complex I dysfunction would phenocopy the 50% reduction of ERG amplitudes that we had observed in global *ndufs4*^*−/−*^ mice by making the RPE more reliant on glucose as a substrate for glycolysis to meet its own energy requirements at the expense of that of the photoreceptors. One possible explanation for the partial effect could simply be inefficiency of tamoxifen-induced Cre-mediated recombination of *floxed ndufs4* in *RPE65-CreER*^*T2*^;*ndufs4*^*loxP/loxP*^ mice. Although we observed a significant reduction of NDUFS4 protein in enriched RPE preparations from *RPE65-CreER*^*T2*^ mice 4 months after tamoxifen induction, it could be that NDUFS4 expression persisted in enough RPE cells to prevent the development of a more pronounced signaling phenotype. Our observation of Cre activity in nearly all RPE cells of *RPE65-CreER*^*T2*^;*Ai9* reporter mice would suggest that Cre activity is quite efficient in the RPE, but it should be noted that complete deletion of the *ndufs4* gene requires Cre-mediated recombination of both *floxed* alleles in each cell. An alternative approach that could circumvent this potential limitation would be to use transgenics to restore *ndufs4* expression specifically in the RPE of global *ndufs4*^*−/−*^ mice and to assess whether NDUFS4-replete RPE partly or completely restore ERG amplitudes to WT levels.

A perhaps more likely explanation for the partial reduction of ERG amplitudes observed in the setting of RPE-specific *ndufs4* deletion is that a more complex outer retinal “metabolic ecosystem” exists in which intact OXPHOS is required in more than just the RPE to create an optimal extracellular environment for photoreceptor signaling. Indeed, it has been reported that disruption of complex IV activity specifically in retinal Müller glia resulted in diminished scotopic ERG a- and b-wave amplitudes similar in proportion to the reductions we observed in the setting of RPE complex I deficiency (34). Therefore, it seems quite plausible that the more dramatic 50% reduction of ERG amplitudes that we observed with global deletion of *ndufs4* may be multifactorial in etiology, resulting from metabolic dysfunction in multiple cell types that collectively establish the extracellular homeostatic conditions of the outer retina. Notably, the close proximity of the glucose transporter GLUT1 on Müller apical processes and photoreceptor inner segments near the external limiting membrane of the retina could provide a direct means for Müller glia to support photoreceptor glycolysis by supplying glucose (10, 35). Deleting *ndufs4* from Müller glia exclusively or in combination with RPE-specific deletion would allow the relative contributions of oxidative metabolism in each type of supporting cell to be determined. Furthermore, although the fenestrated nature of the choriocapillaris allows free diffusion of glucose from the choroidal circulation to the RPE, it remains a consideration that mitochondrial insults within the diverse cell types of the choroid could adversely affect the delivery of metabolic substrates and thereby impact outer retinal function.

A better understanding of the metabolic interplay between photoreceptors and supporting cells in the retina could ultimately lead to therapeutic approaches to improve vision in the setting of chronic pathology. For instance, patients with intermediate age-related macular degeneration often suffer from impaired low-luminance visual acuity that is seemingly out of proportion to the anatomic abnormalities of the retina and RPE (36, 37, 38). It is tempting to speculate that insults to the metabolic ecosystem due to dysfunction of the RPE and/or other cell types may lead to objectively subtle but subjectively impactful changes to vision in these patients. Our prior observation of reversible photoreceptor signaling impairment in the context of global complex I deficiency suggests that interventions that normalize the extracellular milieu of the outer retina might boost the function of otherwise physiologically intact photoreceptors. Targeting the provision of key metabolites to the retina may therefore hold significant promise in optimizing visual function at various stages of retinal disease.

## Experimental procedures

### Animals

*ndufs4*^*loxP/loxP*^ mice with exon 2 of *ndufs4* flanked by loxP sites were obtained from The Jackson Laboratory (stock no. 026963) (15) and crossed with iCre75 mice expressing Cre recombinase under the transcriptional control of the rhodopsin promoter (stock no. 015850) (19) in order to generate *iCre75*;*ndufs4*^*loxP/loxP*^ mice. *ndufs4*^*−/−*^ mice with global deletion of *ndufs4* were also obtained from The Jackson Laboratory (stock no. 027058). *RPE65-CreER*^*T2*^ mice were a generous gift of Dr Philip Kiser (UC Irvine) (29). These were crossed with *Ai9* mice (The Jackson Laboratory stock number 007909) (30) to yield *RPE65-CreER*^*T2*^;*Ai9* reporter mice with tamoxifen-inducible, Cre-dependent tdTomato reporter expression in the RPE. Crosses between the *RPE65-CreER*^*T2*^ and *ndufs4*^*loxP/loxP*^ strains were performed to generate *RPE65-CreER*^*T2*^;*ndufs4*^*loxP/loxP*^ mice. Sequencing of the *rpe65* gene was performed on all strains, and it was confirmed that all were homozygous for the allele encoding the M450 variant of RPE65.

Animals were reared under a normal day/night cycle and handled according to a protocol approved by the Institutional Animal Care and Use Committee of Duke University. Experimental mice carrying *RPE65-CreER*^*T2*^ and control littermates without Cre were similarly administered tamoxifen dissolved in corn oil *via* intraperitoneal injection (75 mg/kg) on postnatal days 22 to 26. All experiments were performed during the day, and mice of both sexes were included in each experimental group.

### Antibodies

The following antibodies were used for western blot analysis of tissue lysates: mouse monoclonal anti-NDUFS4 1-E−4 (1:200; Santa Cruz Biotechnology, sc-100567), mouse monoclonal anti-β-actin (1:1000; Santa Cruz Biotechnology, sc-47778), mouse monoclonal anti-rhodopsin 4D2 (1:5000; Abcam, ab98887), rabbit polyclonal anti-COX4 (1:1000; Abcam, ab16056), rabbit polyclonal anti-Cre (1:10,000; Millipore Sigma, 69050). For immunofluorescence experiments, rabbit polyclonal anti-ZO-1 (1:100; Invitrogen, 40–2200) and rabbit polyclonal anti-Sox9 (1:1000; Sigma-Aldrich, AB5535) were used. Secondary antibodies against the appropriate species conjugated to Alexa Fluor 488 (immunofluorescence experiments, 1:500 dilution) or Alexa Fluor 680 or 800 (western blot experiments, 1:20,000 dilution) were purchased from Invitrogen. Cell nuclei were stained using DAPI (Sigma-Aldrich).

### Electroretinography

ERGs were recorded in live mice of both sexes as described previously using the Espion E3 system with a ColorDome Ganzfeld stimulator (Diagnosys LLC) (14, 39). Briefly, after dark adaptation for 6 h, mice were anesthetized by an intraperitoneal injection of 100 mg/kg ketamine and 10 mg/kg xylazine. Pupils were dilated with a mixture of 0.5% (wt/vol) tropicamide and 1.25% (wt/vol) phenylephrine. A 1% carboxymethylcellulose sodium gel was applied to maintain ocular lubrication during recordings, and body temperature was maintained by a heated platform. Simultaneous recordings were made from both eyes using gold contact lens electrodes (Mayo Corporation), with stainless steel needle electrodes (OcuScience) in the mouth (reference) and at the base of the tail (ground). ERG signals were sampled at 1 kHz and recorded with 0.15 Hz low-frequency and 500 Hz high-frequency cutoffs. Responses to flashes from 0.0001 to 500 cd∗s/m^2^ with 10 to 1 trials averaged and interflash intervals of 5 to 180 s were recorded in the dark. Following the recordings, the mice were euthanized for histological analysis.

The data from the ERG recordings were analyzed as previously described using Matlab R2021b (MathWorks) (14). Oscillatory potentials were removed from the signals by 55 Hz fast fourier transform low-pass frequency filtering. The b-wave amplitude was calculated as the difference from baseline to the peak for dimmer flashes and from the bottom of the a-wave to the b-wave peak for brighter flashes. The c-wave amplitude was calculated as the difference from the preceding trough (immediately following the b-wave peak) to the c-wave peak (in the range of 1–3 s implicit time) as previously described (23). Data points from the a-wave and b-wave stimulus-response curves were fitted by single (Equation 1) or double hyperbolic functions (Equation 2), respectively, using the least-square fitting procedure. *R* is the transient-peak amplitude of the rod response, *R*_*max*_ is the maximal response amplitude, *I* is the flash intensity, *n* is the Hill coefficient (exponent), and *I*_0.5_ is the half-saturating light intensity. Using Prism 9 (Graphpad), *p*-values were calculated to determine the statistical significance between the curve fits in each condition using an F test and between the response amplitudes of the conditional *ndufs4* KO and control mice at each flash intensity using two-tailed *t*-tests.

(1) $$R=R_{\max}\frac{\;I^{n\;}}{I^{n\;}+I_{0.5}^{n\;}}$$

(2) $$R=R_{\max,1}\frac{\;I^{n_{1}}}{I^{n_{1}}+I_{0.5,1}^{n_{1}}}+R_{\max,2}\frac{\;I^{n_{2}}}{I^{n_{2}}+I_{0.5,2}^{n_{2}}}$$

### Histological techniques

To obtain ocular tissue for plastic sections and electron microscopy analysis, mice were euthanized and the superior limbus of each eye marked with cautery to facilitate proper orientation. The eyes were then enucleated and fixed in 2% glutaraldehyde and 2% paraformaldehyde in 0.05% calcium chloride in 50 mM Mops (pH 7.4). Subsequently, 1 μm plastic-embedded cross-sections of the mouse retina were obtained and stained with methylene blue for light microscopy as previously described (40). Photoreceptor nuclei were manually counted within 100 μm segments of the outer nuclear layer spaced at 500 μm steps in cross-sections cut through the optic nerve head. A subset of the same specimens was also processed for transmission electron microscopy. Thin sections of 60 to 80 nm were collected on copper grids, counterstained with uranyl acetate and Sato’s lead, and examined using an electron microscope (JEM-1400; JEOL) at 60 kV. Images were collected using a charge-coupled device camera (BioSprint; Advanced Microscopy Techniques).

To quantify RPE cell survival, eyes were collected from euthanized mice and the anterior segments and retinas promptly removed. The posterior eyecups were placed in PBS and then fixed by incubating in progressively higher concentrations of methanol (50%, 75%, and 87.5%) for 5 min at a time, followed by incubation in 100% methanol for 2 h at room temperature. The fixed eyecups were washed in PBS, blocked in 10% normal donkey serum in PBS with 1% Triton X-100, and immunolabeled with anti-ZO-1 primary antibody and Alexa 488-conjugated secondary antibody. The posterior eyecups were flat mounted on slides with RPE facing up, by making four radial incisions toward the optic nerve and four additional minor radial incisions, followed by mounting under glass coverslips with VECTASHIELD (Vector Laboratories). Images were acquired with a confocal microscope (Eclipse 90i and A1 confocal scanner; Nikon) with a 60 × objective (1.49 NA Plan Apochromat VC; Nikon) using Nikon NIS-Elements software. Image processing was performed with ImageJ and Nikon NIS-Elements software. Briefly, 45,000 μm^2^ images were collected in four quadrants from each flat mount, at two separate distances from the optic nerve head, 500 μm and 1000 μm. Using ImageJ, the number of RPE cells per high power field was counted for each quadrant and averaged for each distance from the optic nerve head.

To quantify Müller glia survival, eyecups were fixed in 4% paraformaldehyde, cryoprotected in 30% sucrose and embedded in optimal cutting temperature medium (Tissue-Tek, Sakura Finetek). Retinal cross-sections of 20-μm thickness were collected through the center of each eyecup using a cryostat (Microm HM 550, Thermo Fisher Scientific). Sections were rehydrated with PBS, blocked with donkey serum, and incubated in anti-Sox9 primary antibody overnight, labeled with secondary antibody and mounted with VECTASHIELD under glass coverslips. Confocal images were acquired using a 60× objective, with three 45,000-μm^2^ images spaced at 500 μm intervals from either side of the optic nerve collected on three sections per retina. Using ImageJ, the number Sox9-positive cell bodies in the inner nuclear layer was divided by the length of inner nuclear layer captured in each image and averaged for each retina.

In all histological experiments, cell counts were compared between experimental groups using Prism 9 software. Two-tailed t tests were used for statistical analysis, with graphs depicting mean ± standard deviation.

### Western blot analysis of retinal and RPE lysates

To prepare lysates for the analysis of the protein content of whole retinas, mice were euthanized and the retinas promptly extracted from the eyes and placed in 100 μl of lysis buffer [25 mM Hepes buffer, pH 7.4, 150 mM NaCl, 5 mM MgCl_2_, and protease inhibitors (cOmplete Mini, Roche) containing 1% Triton X-100]. The retinas were sonicated and centrifuged at 10,000g to clear cellular debris.

For analysis of the protein content of the RPE, an RPE enrichment procedure was performed. Mouse eyes were enucleated and flash frozen in liquid nitrogen. On the day of RPE isolation, eyes were rehydrated in PBS for 10 min. Under a dissection microscope extraocular tissues were cleared and the cornea, lens, and vitreous body were dissected away, followed by careful separation of the retina from the posterior pole avoiding any disturbance to the RPE monolayer. The RPE layer was extracted by gently washing of the posterior cup with PBS using a 100 μl pipette. Any remaining RPE was detached using a paint brush. The PBS solution containing RPE cells was centrifuged at 10,000g for 10 min and the cell pellet resuspended in 35 μl of RIPA extraction buffer containing a protease inhibitor cocktail.

The protein concentrations of each retinal and RPE lysate were determined using bicinchoninic acid (BCA), a colorimetric assay (Bio-Rad). After mixing with SDS-PAGE sample buffer, the retinal and RPE lysates were separated on 4 to 20% SDS-PAGE gels (10 μg of protein per lane), transferred onto polyvinylidene fluoride (PVDF) membranes and blotted with the indicated primary and secondary antibodies. The blots were imaged using an Odyssey imaging system (LI-COR). The band intensity for NDUFS4 was measured for each sample and normalized to that of β–actin. The retinas or RPE of 4 *iCre75*;*ndufs4*^*loxP/loxP*^ mice and *RPE65-CreER*^*T2*^;*ndufs4*^*loxP/loxP*^ mice, respectively, and four of the corresponding control mice were analyzed. Prism 9 software was used to construct 95% confidence intervals for relative NDUFS4 protein abundance for each group, and statistical comparisons were made using two-tailed t tests.

### Serial tangential sectioning of retinal flat mounts

To profile the protein content of different retinal layers, flat-mounted P90 mouse retinas were serially sectioned as previously described (41). Briefly, 2-mm punches of isolated retinas were obtained using a surgical trephine, transferred photoreceptor-side up to a PVDF membrane, flattened between glass slides separated by 0.5 mm spacers, and frozen on dry ice. The flattened retinal specimens were then serially cut into 10-μm-thick sections on a cryostat (CM3050S; Leica), with each section recovered in a separate Eppendorf tube. The tissue sections were dissolved in 40 μl Laemmli buffer, sonicated, and boiled for 10 min. Twenty microliters of each sample was loaded into its own lane for SDS-PAGE. To assess for rhodopsin, 2 μl of each sample was mixed with 18 μl of Laemmli buffer, and then 10 μl of each diluted sample was loaded on a separate gel. After transfer to PVDF membrane, primary antibodies were used to blot for NDUFS4, COX4, actin, and rhodopsin at dilutions described above.

### Complex I enzymatic activity measurements

The activity of respiratory complex I in retinal and RPE/choroid lysates was performed using the Complex I Enzyme Activity Assay Kit (colorimetric) (Abcam, ab109721). For *iCre75*;*ndufs4*^*loxP/loxP*^ mice (n = 7) and *ndufs4*^*loxP/loxP*^ littermates (n = 5), the retinas were removed immediately following euthanasia, and both retinas were pooled and added to 400 μl ice-cold PBS. For the *RPE65-CreER*^*T2*^;*ndufs4*^*loxP/loxP*^ mice (n = 4) and *ndufs4*^*loxP/loxP*^ littermates (n = 4), RPE/choroid tissue was dissected from posterior eyecups, and the tissue from both eyes was pooled in 300 μl ice-cold PBS. Both types of samples were then homogenized with a Dounce homogenizer on ice. Protein concentration of each retinal lysate was determined using a BCA assay (Bio-Rad), and the samples frozen in liquid nitrogen and stored at −80 °C. The abundant RPE pigment would confound the BCA assay, so protein quantification was postponed until clarification of the samples. On the day of the assay, the samples were thawed and prepared according to the manufacturer’s instructions. Briefly, the samples were extracted by adding 1/10 volume of detergent solution for 30 min on ice, followed by centrifugation at 16,000g for 20 min at 4 °C. The supernatants were collected, and at this point protein concentration was measured for RPE samples using a BCA assay. The kinetic assay was performed using final sample concentrations of 470 μg/ml for retina samples and 120 μg/ml for RPE samples.

## Data availability

All relevant experimental data will be made available upon request to the corresponding author, Dr Sidney Gospe (sid.gospe@duke.edu).

## Supporting information

This article contains supporting information.

## Conflict of interest

The authors declare that they have no conflicts of interest with the contents of this article.
